# The Circadian Gene *Per1* Plays an Important Role in Radiation-Induced Apoptosis and DNA Damage in Glioma

**DOI:** 10.31557/APJCP.2019.20.7.2195

**Published:** 2019

**Authors:** Ling Zhu, Qunli Wang, Yi Hu, Fan Wang

**Affiliations:** 1 *Department of Neurosurgery, The First People’s Hospital of Jingmen,*; 2 *Department of Neurosurgery, The Second People’s Hospital of Jingmen, Jingmen, China. *

**Keywords:** Glioma- per1- radiotherapy- apoptosis- DNA damage

## Abstract

**Objective:**

*Period1 (PER1)*, a core circadian gene, not only modulates circadian rhythm but may also play an important role in other biological processes, including pathways involved in the proliferation and apoptosis of tumor cells. In this study, we investigated the mechanism by which the downregulated expression of *PER1* promotes the apoptosis of wild-type *P53* human glioma U343 cells exposed to X-rays.

**Methods:**

U343 cells were exposed to 6 mV 10 Gy X-ray irradiation after infection with an shRNA lentivirus to reduce the expression of PER1 and were analyzed by SCGE analysis, flow cytometry, qRT-PCR, and western blotting.

**Result:**

SCGE analysis revealed that compared with the controls, U343 cells expressing low levels of PER1 showed minor DNA damage when exposed to X-ray irradiation (P<0.05), and the flow cytometry assay showed lower death rates (P<0.05). RT-PCR and western blot analysis both revealed decreased expression of *CHK2* and *P53*, which regulate DNA damage and repair via the CHK2-P53 pathway, and decreased expression of *C-MYC*, which is related to cell apoptosis.

**Conclusion:**

Our research suggests that *PER1 *may play an important role in tumor radiotherapy, which is attributable to enhanced *chk2-P53 *signaling and proapoptotic processes. These findings provide a new target for the clinical treatment of glioma and a reliable basis for postradiation therapy and gene therapy for glioma and other cancers.

## Introduction

The clinical management of malignant glioma is one of the most difficult treatments faced by the practicing neurologist. Patients have a very poor prognosis, largely because of the paucity of druggable targets responsive to therapeutic intervention. However, recent progress at the molecular level has identified candidate targets, many of which have the potential to become future treatment options.

The *Period1 (PER1)* gene is an indispensable component of the mammalian circadian clock (Zheng et al., 2001). In 2002, the murine *PER1* gene was reported to play an important role in tumorigenesis. A higher incidence of tumor development was observed in *PER1*-deficient mice than in wild-type mice (Yang et al., 2009). In addition, clear differences in *PER1 *expression are evident in tumor tissues and noninvolved peripheral tissues (Kolomeichuk et al., 2011; Savvidis et al., 2012; Winter et al., 2007; Xia et al., 2010; Gery et al., 2006). Elsewhere, lowered expression and/or mutations in the *PER1* gene have been reported to correlate with enhanced tumor growth in breast cancer, colon cancer and lymphoma, corresponding with altered expression of *P53* and the oncogenes *BCLxl*, *BCL-2*, *cyclinB1*, *cyclin D*, *cyclin E* and *C-MYC *(Mostafaie et al., 2009; Pluquet et al., 2013; Sato et al., 2011; Ye et al., 2015). *PER1* has also been linked with DNA damage response pathways (Fu et al., 2016). X-ray-induced DNA damage in tumor cell lines with mutated *PER1* exhibited an increased sensitivity to damage affecting cell proliferation, apoptosis, and the *P53* pathway (Yang et al., 2009; Hong et al., 2009). In addition, scholars have concluded that manipulation of Per1 levels influences both the transcription of a broad range of *p53* target genes and the stability of endogenous *p53* (Li et al., 2016). Simultaneously, they also established that *Per1* directly acts on the *p53* node, as checkpoint components upstream of p53 remained active in response to DNA damage. Quantitative transcriptional analyses of *p53* target genes demonstrated that unbound p53 was absolutely required for the activation of the DNA-damage response (Dakup et al., 2016; McKenna et al., 2012).

While it is evident that *PER1* is critical to cell proliferation, apoptosis and the *P53* pathway in neoplasia, although the mechanism remains poorly understood. Based upon previous studies, we examined the role of *PER1* in the cellular response to X-ray-induced DNA damage in the human glioma U343 cell model. RNA interference technology was used to downregulate the expression of *PER1*. We observed that U343 cells with low-level expression of *PER1* exposed to low-dose X-ray irradiation exhibited minor DNA damage and a decreased death rate. Our data indicate that *PER1* interacts with the *P53* pathway and contributes to increased *P53* activity, protecting DNA-damaged cells from *P53*-mediated apoptosis.

## Materials and Methods

*Cell lines and reagents*

The U343 glioma cell line was purchased from Ji Ni Biotechnology Co. Ltd. (Guangzhou, China). Cells were maintained according to the supplier’s instructions and guidelines and were cultivated at 37°C in a 100% RH, 5% CO_2_, 95% air atmosphere. ShRNA-PER1 lentivirus was obtained from Genechem Chemical Technology Co. Ltd. (Shanghai, China). Oligonucleotide primer and probe sequences were designed and provided by Sangon Biotech Co. Ltd. (Shanghai, China). The Annexin V-FITC Apoptosis Detection Kit (Invitrogen, USA), Comet Assay for DNA Damage Detection Kit (KeyGEN Biotechnology, China), puromycin (Solarbio Technology Co. Ltd., China), and Maxima SYBR Green qPCR Master Mix (Fermentas, USA) were used according to the manufacturers’ instructions.

*Transfection with shRNA lentivirus*

The expression of PER1 was regulated using lentiviral transfection of shRNA. U343 glioma cells were stably transformed with shRNA-PER1 and shRNA-control constructs according to the manufacturer’s protocol. U343 cells were plated in 6-well plates at a density of 2×10^4 ^cells/well in RPMI-1640 medium (Gibco, USA) and allowed to attach for 18 h prior to transfection with shRNA lentivirus vectors. Cells were permitted to integrate for 24 h after infection. Puromycin (3 µg/ml) was added to select cells with the integrated retrovirus, and stable cell lines were established after one week. Interference sequences of *shRNA-PER1* are shown as follows.

*X-ray irradiation*

The three above-mentioned cell groups were exposed to 12.4 Gy/min, an exposure field of 25×25 cm^2^ and spacing of 80 cm, and an accumulated dose of 10 Gy X-rays (Varian Medical Systems Inc., California, USA).

**Figure 1:**
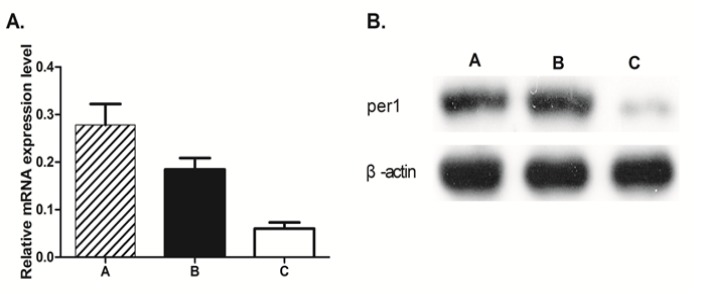
Expression of *Per1 mRNA* and *Per1* Protein in U343 Human Glioma Cell Lines. Figure 1A, U343 cells expressing *shRNA*-control and *shRNA-per1* were detected by real-time PCR. Figure 1B, Total cellular protein was isolated from U343 glioma cells, and 40–60 µg was separated electrophoretically, transferred to nitrocellulose and subjected to western blot analysis with antibodies directed against the per1 proteins. U343 cells were cultivated in vitro under standard conditions. (A) *TPA* alone; (B) *TPA* and *shRNA*-control; and (C) TPA and* shRNA-PER1*

**Figure 2:**
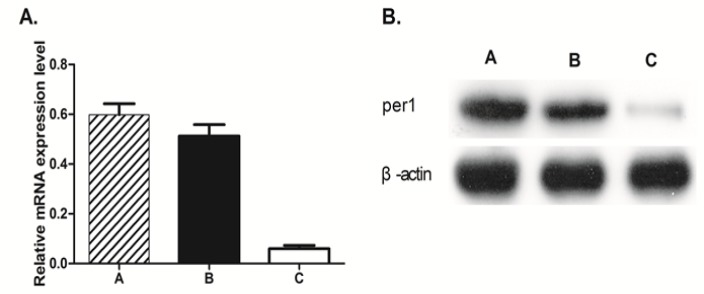
Effect of X-ray Irradiation on the Expression of Per1 in U343 Human Glioma Tumor Cells. Figure 2A, Relative mRNA levels of per1 in U343 cells transfected with shRNA-control or shRNA-per1 were measured by QRT-PCR. Figure 2B, Total cellular protein was isolated from U343 human glioma cells before and after exposure to X-ray irradiation. Then, 40–60 µg of total cellular protein was electrophoretically separated, transferred to nitrocellulose and subjected to western blot analysis using antibodies directed against PER1 protein. β-actin was used as a loading control. (A) TPA alone; (B) TPA and shRNA-control; and (C) TPA and shRNA-PER1

**Figure 3:**
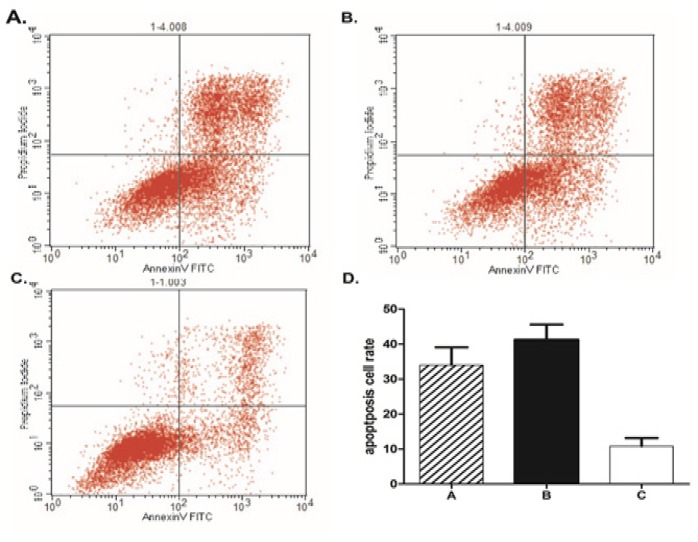
X-ray Irradiation Increases Apoptosis in U343 Glioma Cells after the Downregulation of PER1. Following treatment with irradiation and staining with Hoechst 33,342, cells were subjected to flow cytometry analysis to determine the proportion of apoptotic cells. (A) TPA alone; (B) TPA and shRNA-control; and (C) *TPA* and *shRNA-PER1. *TPA indicates tissue plasminogen activator

**Figure 4:**
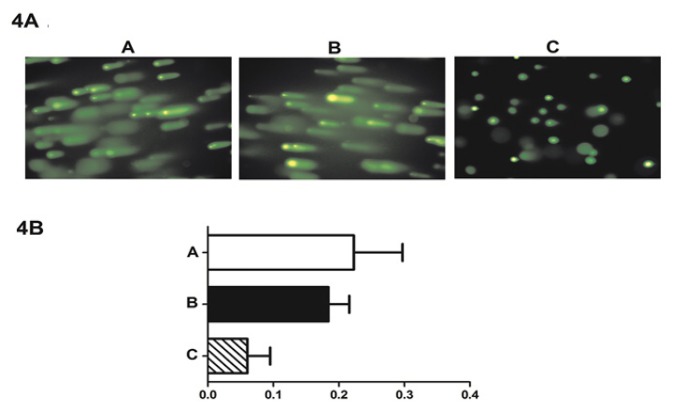
Single-Cell Gel Electrophoresis (SCGE) Analysis of X-ray (10 Gy)-Induced DNA Damage in U343 Cells.DNA damage repair capacity assessed via the comet assay.(A) TPA alone; (B) TPA and shRNA-control; and (C) TPA and shRNA-PER1.This response was statistically significant (P<0.05; n=3)

**Figure 5:**
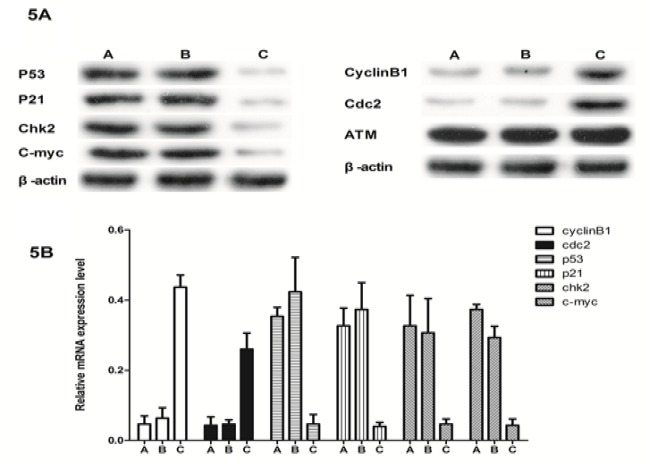
Effect of sh-RNA-per1 on ATM, TP53 and C-MYC mRNA and Protein Expression in U343 Human Glioma Tumor Cell Lines Following Irradiation. Figure 5A,Relative mRNA levels were determined by qRT-PCR. Data are expressed as ΔΔCT relative to the control.Figure 5B, Total U343 glioma cell extracts were examined by quantitative western blotting at 24 h after exposure to 10 Gy X-ray irradiation. Antibodies were specific for ATM, CHK2, p21, P53, cyclinB1, cdc2, and C-MYC . (A) TPA alone; (B) TPA and shRNA-control; and (C) TPA and shRNA-PER1

*Comet-FISH and apoptosis assay*

The alkaline comet assay was performed following the standard protocol of McKelvey-Martin et al., (2012). U343 glioma cells were harvested and washed twice with phosphate buffered saline (PBS) and mixed with 0.7% low-melting point agarose. An agarose gel sandwich was prepared comprising a base layer of 0.5% standard agarose and a second layer of 0.7% low-melting point agarose with cells and overlaid with 0.7% low-melting point agarose to a depth of 0.5 mm. The assay was incubated at 4°C in a high humidity environment for 30 min. Gels were immersed in alkaline lysis solution (90% lysis buffer pH 10 and 10% DMSO) and maintained in the dark for 2 h at 4°C. Gels were briefly rinsed with PBS, equilibrated in 300 mM NaOH and 1 mM EDTA (pH 13) for 40 min, and then subjected to 100 mA at 25 mV for 30 min. Gels were stained with propidium iodide, and DNA comet tails were visualized with a fluorescence microscope at an excitation wavelength of 530 nm and emission wavelength of 620 nm. Apoptosis analysis was performed with the Annexin V-FITC Apoptosis Detection Kit I (BD PharMingen, San Diego, CA) according to the manufacturer’s instructions. Cells were analyzed using a flow cytometer (excitation 488 nm; emission 525 nm (GFP) and 620 nm (PI)) (BD Bioscience, USA).

*Real-time PCR*

Gene expression was quantified using real-time PCR analysis and SYBR Green (Fermentas, USA). Total RNA was extracted from U343 cells with TRIzol and reverse-transcribed into cDNA using a RevertAid First Strand cDNA Synthesis Kit (Fermentas, USA). Samples were analyzed in triplicate using a CXF96 Real-time PCR System (Bio-Rad, Hercules, Ca, USA). A total of 500 ng of sample cDNA was subjected to the following program: 2 min at 50°C (annealing), 10 sec at 95°C (melting), followed by 40 cycles of 15 sec at 95°C, 30 sec at 56°C and a 30 sec extension at 72°C.

*Western blotting*

U343 glioma cells were harvested, and total protein was extracted at 24 h after X-ray irradiation. Isolated proteins (40–60 mg) were separated using SDS–PAGE and transferred to nitrocellulose membranes (Millipore, USA). Nonspecific binding sites were blocked with albumin, and membranes were probed with antibodies specific for *ATM*, *CHK2*,* p21*,* P53*,* cyclinB1*, *cdc2*, and *C-MYC* (Abcam, USA). Each assay was repeated at least three times. Images and relative expression levels of target genes were captured and quantified using Gel-Pro Analyzer (Media Cybernetics, USA).

*Statistical Analysis*

Data are presented as the mean ± SD, and Student’s t-test was used for comparisons among different groups. P-values of less than 0.05 were considered statistically signiﬁcant.

## Results

*Expression of PER1 in U343 glioma cells*

Quantitative real-time PCR (qRT-PCR) and western blotting were applied to analyze PER1 expression at the RNA and protein levels, respectively. Both RNA and immunoreactive proteins were notably reduced in U343 cells transfected with *shRNA-PER1* relative to shRNA-transfected control U343 cells (P<0.05; n=3) (Figure 1). The expression level of *PER1 mRNA* was consistently reduced by more than 70% in *shRNA-PER1 *cells (Figure 1A). Cells transfected with *shRNA-PER1 *also expressed reduced PER1 protein levels compared with the control cells (Figure 1B).

Having demonstrated that lentiviral-mediated RNA interference technology was an effective inhibitor of PER1 expression, subsequent experiments utilized this technology to examine the role of *PER1* in glioma cells subjected to low-dose X-ray irradiation.

*X-ray irradiation enhances PER1 expression in U343 glioma cells*

This experiment was intended to study the role of PER1 in apoptosis induced by X-ray irradiation. Prior to the start of the experiment, we verified whether there was a relationship between* PER1* and X-ray radiation. We measured the expression of *PER1* before and after irradiation. *PER1* expression increased, both at the mRNA level (qRT-PCR) and at the protein level (western blot) (Figure 2).

*Downregulation of PER1 decreases apoptosis in X-ray-irradiated U343 glioma cells*

To determine the possible effects of irradiation on the apoptosis of glioma cells, U343 cells were double-stained with Annexin V-FITC and propidium iodide and analyzed using flow cytometry. The proportion of apoptotic cells in the TPA+shRNA-per1 group (11.29%) after irradiation treatment was significantly lower than that in the TPA alone group (33.46%, P<0.01) and the TPA+ control group (41.71%, P<0.01). These data suggested that the downregulation of *PER1 *was associated with a significant decrease in apoptosis of the U343 cell population after exposure to X-ray ionizing radiation.

*Downregulation of PER1 attenuates radiosensitivity and DNA damage in U343 glioma cells*

After exposure to 10 Gy of X-ray radiation, U343 cells were selected with puromycin for 7 days prior to analysis for DNA damage. Single cell gel electrophoresis (SCGE) revealed the presence of differential DNA damage. We noted that DNA tailing was less prominent in cells transfected with shRNA-PER1 compared to the group transfected with shRNA-control+TPA and the TPA alone group. The tail moment was 0.251 + 0.062 for the TPA alone group, 0.191 + 0.023 for the negative control siRNA group, and 0.093+0.001 for the targeted siRNA group (P<0.05) (Figure 4). These data indicated that the downregulation of PER1 decreased glioma cell sensitivity to ionizing radiation, resulting in decreased DNA damage.

*Effects of siClock on cellular apoptosis-related Genes*

Because our data suggested that *PER1* positively influenced the programmed cell death of U343 glioma cells, we subsequently examined the expression of the following genes known to be important in DNA damage repair and programmed cell death: *ATM*,* CHK2*,* p21*,* P53*,* cyclinB1*,* cdc2, *and* C-MYC.* As shown in Figure 4, shRNA treatment resulted in a clear reduction in the expression of the per1 protein compared to the control treatment. Moreover, the protein expression of *p53*, *p21* and *chk2* were downregulated, and the expression of *cdc2* and *Cyclin B1* were upregulated by *shRNA-per1* treatment (Figure 5A and B). However, the protein expression levels of c-myc and ATM were not significantly altered by siPER1 treatment.

## Discussion

In recent years, research on the core clock circadian gene *PER1* has shown that this gene maintains the biological clock and a 24 h oscillation. Reduced levels of Per1 and the disruption of the 24 h circadian rhythm were found in liver cancer, breast cancer, colon cancer, lymphoma, and glioma patients. We have investigated the role of *PER1* in glioma cells. Evidence from initial research implicated* PER1* as a regulator of the circadian clock(Han et al., 2016; Korge et al., 2018; Repouskou et al., 2016; Reinhardt et al., 2012; Riley et al., 2008); however, recent evidence has expanded the function of PER1 to include the promotion of apoptosis in cancer cells (Han et al., 2016; Korge et al., 2007). We hypothesized that *PER1* is also a tumor suppressor gene.

Experimental evidence from our previous studies indicated that the expression of *PER1* and *PER2* closely correlated with the incidence and development of glioma. Moreover, biological rhythms have also become an important prognostic indicator of the survival of glioma patients. However, our previous data from C6 glioma cells studied in vitro also indicated their involvement in biological rhythms. Following the administration of radiation at different time points, cells expressing high levels of *PER1* showed a death rate significantly higher than those of other cells. Similar results could be observed in other tumors. In nude mice subcutaneously injected with human tongue cancer cells,* PER1* knockdown in the cells enhanced tumor development, leading to increased tumor weights and volumes. All of the above data suggest that *PER1* plays an important role in promoting apoptosis in cancer cells. These findings are consistent with our data from human glioma cancer specimens.

Apoptosis was induced by X-ray after DNA damage. When DNA damage occurs, it continues to activate several checkpoints, such as *ATM/ATR*, *CHK1/2*, and so on. Then, *P53*, an important protein in the regulation of DNA damage and repair and the cell cycle, is activated (Korge et al., 2007; Orren et al., 1997 ), which results in the arrest of the cell cycle at the *G1/S*, *S* or *G2/M* phase to provide sufficient time for the repair of DNA damage (Weinert et al., 1997). If DNA damage is not properly repaired, chromosomal deletions, duplications or translocations can occur; thus, the P53 protein will instantly activate *P21 (CDKN1A)*, *BAX* and other related genes to prevent gene mutation and carcinogenesis (Korge et al., 2007; Weinert et al., 1997). In addition, P53 is also connected to promoter binding of the oncogene *C-MYC *and inhibits its transcription by promoting deacetylase (Ho et al., 2005). Conversely, P53 in the cytoplasm can directly act on proapoptotic BCL family members to enhance the transduction of apoptotic signals (Ho et al., 2005).

In this study, we focused on the role of *PER1* in DNA damage, apoptosis and radiosensitivity of glioma cells induced by X-ray irradiation. After comprehensively surveying the links between *PER1* and *P53* in previous reports[2, 4, 9-14], we selected wild-type P53 human U343 glioma cells. Then, RNA interference technology and *shRNA* lentivirus were used to downregulate *PER1*. However, we found that following treatment with X-ray irradiation, DNA damage was observed in the TPA+shRNA-control group, and the TPA alone group cells showed more serious DNA tailing phenomena and a significantly increased death rate compared with those in the *TPA+shRNA-per1* group. Following exposure to X-ray irradiation, U343 cells with high *PER1* expression levels had serious DNA damage and a higher death rate. We mainly focused on the mechanism by which *PER1* regulates the apoptosis of U343 cells through the ATM–P53 pathway to initiate DNA damage repair. The important ATM checkpoint in DNA damage should be activated when exposed to X-ray irradiation and start the DNA repair mechanism mediated by P53 to ensure the maximum repair of damaged DNA. However, in our research, the expression of ATM was not significantly different in U343 cells with low *PER1* expression levels compared to other cells when exposed to X-ray irradiation, causing a significantly different expression of P53. This shows that in addition to the effect of X-ray irradiation, ATM can also be affected by other factors, such as *per1*, which has an important direct impact. 

*CHK2*, which is involved in DNA repair, cell cycle arrest or apoptosis in response to DNA damage, was significantly lower in the *TPA+shRNA-per1* group than in the TPA alone and TPA shRNA-control group. By keeping other disruption factors consistent, we found that the downregulation of the expression of *PER1* can decrease the expression of activated CHK2 and P53. Therefore, we hypothesized that it is not only involved in the regulation of DNA damage but also regulates cell apoptosis via the CHK-P53 pathway as the upstream gene of *chk2* and *P53*.

The tumor suppressor* p53 *is a transcription factor that is activated in response to DNA damage or oncogenic transformation. The loss or mutation of* p53* in many cancers leads to impaired cell cycle regulation, genomic instability, and the inhibition of apoptosis The protein levels of *p53* in the *per1*-silenced cells tended to be lower than those in the control cells, indicating that *per1* regulated *p53* expression, and this activity may at least partially contribute to the apoptosis of *per1*-silenced cells. In the present study, the expression levels of *cdc2* and *cyclin B1 *were upregulated by shRNA-*per1* treatment. Our findings support previous studies that showed that p53 regulates *Cdc2* and *Cyclin B1* through transcriptional suppression. We also speculate that the *per1* gene is involved in the regulation of *cdc2* and *cyclinB1* through the regulattion of *P53*.

The circadian clock-controlled gene c-myc plays a key role in cell proliferation and apoptosis. The deregulation of c-myc has been associated with various cancers and with the hyperplastic growth of mammalian tissues. In our study, we focused on examining circadian clock-regulated gene expression to emphasize the fundamental role that the circadian clock plays in glioma cell apoptosis following irradiation. We found that the levels of c-myc were significantly decreased in irradiated* shRNAper1*-transfected glioma cells and that c-myc overexpression induced genomic DNA damage and compromised *p53* function, presumably via a reactive oxygen species-mediated mechanism.

These results indicated the important effect of *PER1* on the apoptosis of U343 cells caused by irradiation. On the one hand, downregulation of *PER1* can worsen the radiosensitivity of U343 glioma cells, while the expression of important checkpoints in DNA damage, such as* CHK2 *and *P53*, are decreased by the regulation of *PER1*, which reduces the effect of the *chk2–P53* pathway activated by DNA damage. Moreover, C-MYC is also controlled by the circadian clock (Kolomeichuk et al., 2011); together with the two proteins described above, C-MYC can induce apoptosis in U343 cells.

In conclusions, it can be concluded from our present study that the downregulation of *PER1* can decrease the sensitivity of U343 glioma cells exposed to X-ray irradiation and reduce apoptosis. The mechanism by which it does this can be summarized as changes in the expression of *PER1*, which can activate the *CHK2–P53 *pathway and other genes associated with cell cycle arrest and apoptosis caused by X-rays, such as *c-myc*, *P53*, *p21*, *cdc2* and *cyclineB1*, thus further inducing apoptosis. Therefore, we hypothesize that *PER1*, the core circadian gene, is not only a tumor suppressor gene but can also be regarded as an upstream regulatory gene of* P53*, which plays an important role in inhibiting tumor growth and promoting apoptosis of cancer cells by regulating *P53 *expression, DNA damage repair and apoptosis. These findings provide a new target for the clinical treatment of glioma and a reliable basis for postradiation therapy and gene therapy for glioma and other cancers.

## Funding Statement

Supported by Hubei Provincial Nature Science Foundation grant: 2017CFC823.

## References

[B1] Dakup P, Gaddameedhi S (2017). Impact of the Circadian clock on UV-induced DNA damage response and photocarcinogenesis. Photochem Photobiol.

[B2] Fu XJ, Li HX, Yang K (2016). The important tumor suppressor role of PER1 in regulating the cyclin-CDK-CKI network in SCC15 human oral squamous cell carcinoma cells. Onco Targets Ther.

[B3] Gery S, Komatsu N, Baldjyan L (2006). The circadian gene per1 plays an important role in cell growth and DNA damage control in human cancer cells. Mol Cell.

[B4] Han Y, Meng F, Venter J (2016). MiR-34a-dependent overexpression of Per1 decreases cholangiocarcinoma growth. J Hepatol.

[B5] Ho JS, Ma W, Mao DY, Benchimol S (2005). p53-Dependent transcriptional repression of c-myc is required for G1 cell cycle arrest. Mol Cell Biol.

[B6] Hong CI, Zámborszky J, Csikász-Nagy A (2009). Minimum criteria for DNA damage-induced phase advances in circadian rhythms. PLoS Comput Biol.

[B7] Kolomeichuk SN, Gurov EV, Piskunova TS, Tyndyk ML, Anisimov VN (2011). Expression of circadian Per1 and Per2 genes in the liver and breast tumor tissues of HER2/neu transgenic mice of different age. Bull Exp Biol Med.

[B8] Korge S, Maier B, Brüning F (2018). The non-classical nuclear import carrier Transportin 1 modulates circadian rhythms through its effect on PER1 nuclear localization. PLoS Genet.

[B9] Li HX, Fu XJ, Yang K (2016). The clock gene PER1 suppresses expression of tumor-related genes in human oral squamous cell carcinoma. Oncotarget.

[B10] McKenna DJ, Doherty BA, Downes CS (2012). Use of the comet-FISH assay to compare DNA damage and repair in p53 and hTERT genes following ionizing radiation. PLoS One.

[B11] Mostafaie N, Kállay E, Sauerzapf E (2009). Correlated downregulation of estrogen receptor beta and the circadian clock gene Per1 in human colorectal cancer. Mol Carcinog.

[B12] Orren DK, Petersen LN, Bohr VA (1997). Persistent DNA damage inhibits S-phase and G2 progression, and results in apoptosis. Mol Biol Cell.

[B13] Pluquet O, Dejeans N, Bouchecareilh M (2013). Posttranscriptional regulation of PER1 underlies the oncogenic function of IREα. Cancer Res.

[B14] Reinhardt HC, Schumacher B (2012). The p53 network: cellular and systemic DNA damage responses in aging and cancer. Trends Genet.

[B15] Repouskou A, Prombona A (2016). c-MYC targets the central oscillator gene Per1 and is regulated by the circadian clock at the post-transcriptional level. Biochim Biophys Acta.

[B16] Riley T, Sontag E, Chen P, Levine A (2008). Transcriptional control of human p53-regulated genes. Nat Rev Mol Cell Biol.

[B17] Sato F, Wu Y, Bhawal UK (2011). PERIOD1 (PER1) has anti-apoptotic effects, and PER3 has pro-apoptotic effects during cisplatin (CDDP) treatment in human gingival cancer CA9-22 cells. Eur J Cancer.

[B18] Savvidis C, Koutsilieris M (2012). Circadian rhythm disruption in cancer biology. Mol Med.

[B19] Vousden KH, Lane DP (2007). p53 in health and disease. Nat Rev Mol Cell Biol.

[B20] Weinert T (1997). A DNA damage checkpoint meets the cell cycle engine. Science.

[B21] Winter SL, Bosnoyan-Collins L, Pinnaduwage D, Andrulis IL (2007). Expression of the circadian clock genes Per1and Per2 in sporadic and familial breast tumors. Neoplasia.

[B22] Xia HC, Niu ZF, Ma H (2010). Deregulated expression of the Per1 and Per2 in human gliomas. Can J Neurol Sci.

[B23] Yang X, Wood PA, Ansell CM (2009). The circadian clock gene Per1 suppresses cancer cell proliferation and tumor growth at specific times of day. Chronobiol Int.

[B24] Ye H, Yang K, Tan XM (2015). Daily rhythm variations of the clock gene PER1 and cancer-related genes during various stages of carcinogenesis in a golden hamster model of buccal mucosa carcinoma. Onco Targets Ther.

[B25] Zheng B, Albrecht U, Kaasik K (2001). Nonredundant roles of the mPer1 and mPer2 genes in the mammalian circadian clock. Cell.

